# CAISC: A software to integrate copy number variations and single nucleotide mutations for genetic heterogeneity profiling and subclone detection by single-cell RNA sequencing

**DOI:** 10.1186/s12859-022-04625-x

**Published:** 2022-03-21

**Authors:** Jeerthi Kannan, Liza Mathews, Zhijie Wu, Neal S. Young, Shouguo Gao

**Affiliations:** Hematopoiesis and Bone Marrow Failure Laboratory, Hematology Branch, NHLBI, National Institutes of Health, Bethesda, MD 20892 USA

**Keywords:** Single-cell RNA sequencing, Copy number variation, Single nucleotide variation, Entropy-based weighted integration

## Abstract

**Background:**

Although both copy number variations (CNVs) and single nucleotide variations (SNVs) detected by single-cell RNA sequencing (scRNA-seq) are used to study intratumor heterogeneity and detect clonal groups, a software that integrates these two types of data in the same cells is unavailable.

**Results:**

We developed Clonal Architecture with Integration of SNV and CNV (CAISC), an R package for scRNA-seq data analysis that clusters single cells into distinct subclones by integrating CNV and SNV genotype matrices using an entropy weighted approach. The performance of CAISC was tested on simulation data and four real datasets, which confirmed its high accuracy in sub-clonal identification and assignment, including subclones which cannot be identified using one type of data alone. Furthermore, integration of SNV and CNV allowed for accurate examination of expression changes between subclones, as demonstrated by the results from trisomy 8 clones of the myelodysplastic syndromes (MDS) dataset.

**Conclusions:**

CAISC is a powerful tool for integration of CNV and SNV data from scRNA-seq to identify clonal clusters with better accuracy than obtained from a single type of data. CAISC allows users to interactively examine clonal assignments.

**Supplementary Information:**

The online version contains supplementary material available at 10.1186/s12859-022-04625-x.

## Background

Cancer progression involves successive waves of clonal selection within the tumor. Mutations that increase fitness within the local environment are likely to drive clonal expansion, leading to competition and coexistence of clones and tumor heterogeneity, both in clone composition and clinical phenotypes. In order to diagnose cancer and determine potential therapeutic targets, it is important to quantify this intra-tumor heterogeneity by identifying the subgroups of cells (or subclones) that survive as a tumor undergoes evolution [1]. Reconstructing phylogenies from bulk tumor samples is difficult, since they contain mixtures of mutations from many heterogeneous cells [2, 3]. Single-cell RNA sequencing (scRNA-seq) addresses this issue by allowing detection of mutations in expressed genes when they are present in individual single cells. More importantly, co-occurrence patterns among mutations across multiple single cells can help define sub-clonal populations and elucidate evolutionary dynamics [2, 4–7]. However, single cell sequencing data has high rates of amplification and sequencing errors.

Somatic DNA alterations in tumors range the genomic scales, including single nucleotide variations (SNVs), copy-number variations (CNVs), and aneuploidy. Presently, subclone identification studies mainly assess SNV. There are several limitations to this approach with SNV. For example, only a small portion of the SNVs of each cell is expected to be derived by the reads of scRNA-seq, meaning that only SNVs in a transcribed region are covered [8]. The coverage at the 5’ end is very low, and most of the reads are located on the 3’ end in the data of Smart-seq. If a mutation is located on the 5’ end, it is difficult to be captured. Therefore, we consider the gene body coverage to be poor [9, 10]. As a result SNV-based subclone detection with scRNA-seq is difficult. Only some SNVs in each cell are expected to be visible in the scRNA-seq read output; in order to be sequenced and observed, the SNV must occur in transcribed regions of the genome. Even if the SNV satisfies this condition, the mutated alleles are often missing due to biological or technical dropout. Because of the “burst” nature of gene transcription, large fractions of genes are only expressed from one of the alleles at any given time, and hence an SNV residing in a gene that is expressed at the bulk tissue levels may not be observed in a cell due to chance, a phenomenon known as biological dropout. Additionally, a mutated allele that is expressed must be successfully converted to cDNA in preparation for sequencing in order to be detected, and absence of such alleles is denoted technical dropout. Finally, post-transcriptional modification, low sequencing depth, and sequencing errors also impact the sensitivity and specificity of SNV discovery [8].

There have been many models used in single-cell phylogenetic analysis that allow missing values of SNVs [1]. Both scSNV and scCNV play important roles in tumor generation and progression in cancer and contribute to tumor heterogeneity [2, 5, 11, 12]. A model that incorporates both SNV and CNV data for inference of phylogenetic structures would potentially provide more accurate reconstructions of single-cell tumor phylogenies [8]. Previous studies have shown that joint analysis of scDNA-seq and scRNA-seq enables cell-resolved investigation of pathological tissue clones [13, 14]. Clonal definitions with only one type of marker may lead to loss of detail [1]. We illustrate in Additional file 1: Fig. S1C: subclone A is located at the root, and other subclones result from point mutations and CNVs. The two SNVs give rise to subclones B and D, and the two CNVs give rise to subclones C and E. If we infer the clone tree using only SNV data, we generate a linear evolutionary history, without information of copy loss and gain (Additional file 1: Fig. S1B). Therefore, clone C cannot be distinguished from clone A as they have the same SNV profile (Additional file 1: Fig. S1A). Similarly, clone B cannot be distinguished from clone E. In this case, complete ontogeny can only be reconstructed with both SNV and CNV profiles.

**Fig. 1:**
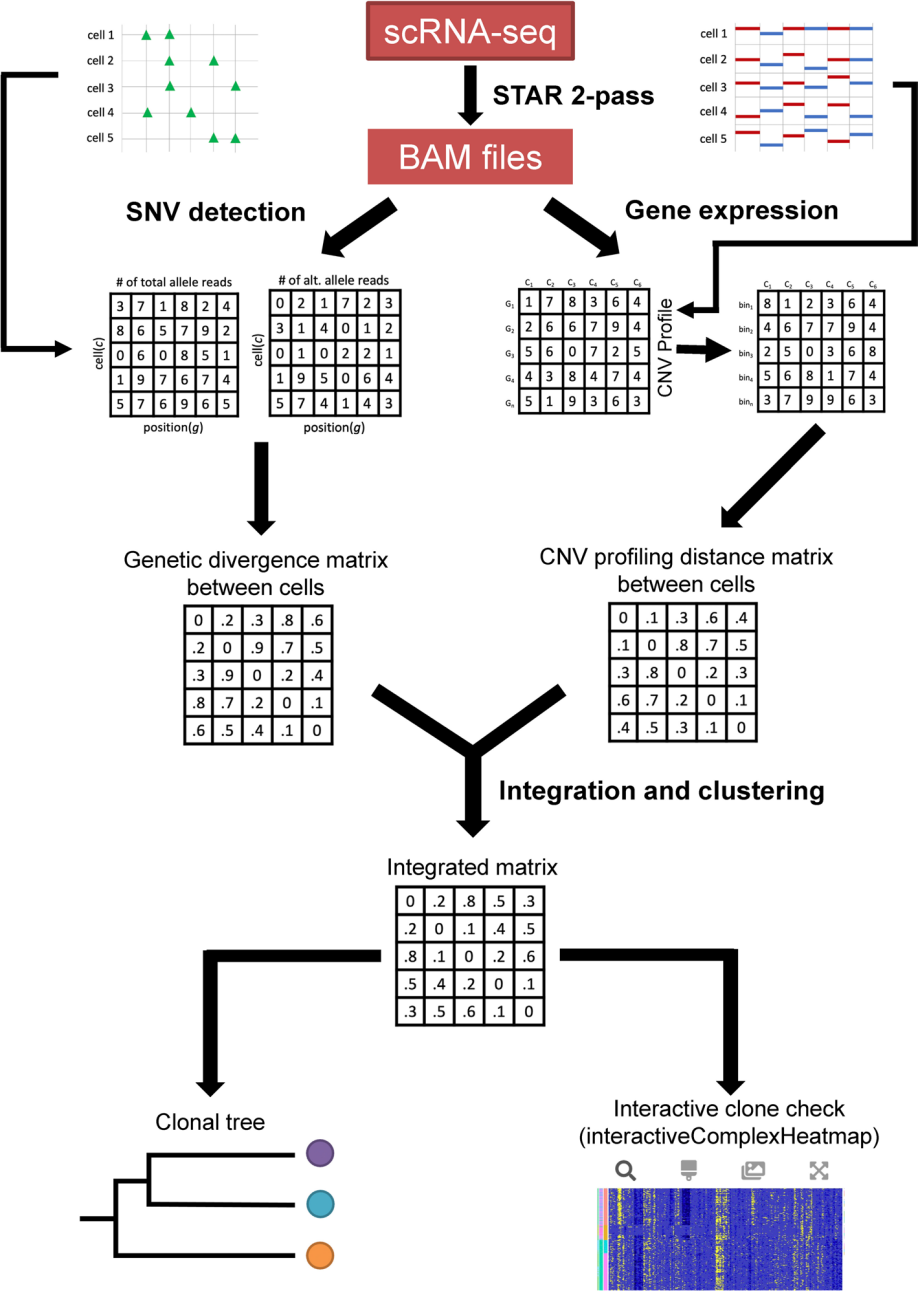
Clonal Architecture with Integration of SNV and CNV (CAISC). Overview of the computational framework that integrates both SNV and CNV profiles for clonal identification from scRNA-seq data. scRNA-seq reads are aligned with STAR, mutations are identified with GATK, and the read counts were calculated to represent gene expression. DENDRO is used to calculate a distance matrix based on allele reads of mutations. Infercnv is used to convert gene expression level data to a CNV profile matrix and then to calculate a distance matrix between cells. These two distance matrices are integrated using an entropy weighted method. Finally, a clonal tree is generated using the integrated matrix to more accurately identify subclones and infer their evolutionary relationship. The interactive figure generated by interactiveComplexHeatmap allows for manual examination of inferred clones

To the best of our knowledge, SCARLET (single-cell algorithm for reconstructing loss-supported evolution of tumors) is the only method that integrates SNV and CNV data from single cell DNA-seq [1]. SCARLET builds a coarse phylogenetic tree with CNV data alone, and SNV data are employed to refine the tree. However, SCARLET does not utilize a unified evolutionary model for both SNV and CNV data. Instead it provides a way to directly integrate SNVs with prior evolutionary CNV models, which could result in lower quality measurements of SNVs and CNVs. Here, we present a new algorithm termed as Clonal Architecture with Integration of SNV and CNV, or CAISC, which allows subclone detection by integration of both SNV and CNV data and generates more accurate and robust clustering results (Fig. 1). We derive two cell–cell distance matrices using SNV and CNV data, from DENDRO [8] and infercnv [7], respectively. These matrices are integrated using an entropy weighted method into a final distance matrix that is used to cluster the cells into subclones. Using the adjusted rand index (ARI), we evaluated the CAISC approach against other SNV-based and CNV-based approaches. The CAISC package, implemented in R, is available at https://github.com/lizamathews/CAISC, where we also provide source code and sample datasets.

## Results

### Cells in clones defined by SNV tend to have similar CNV profiles

**Fig. 2:**
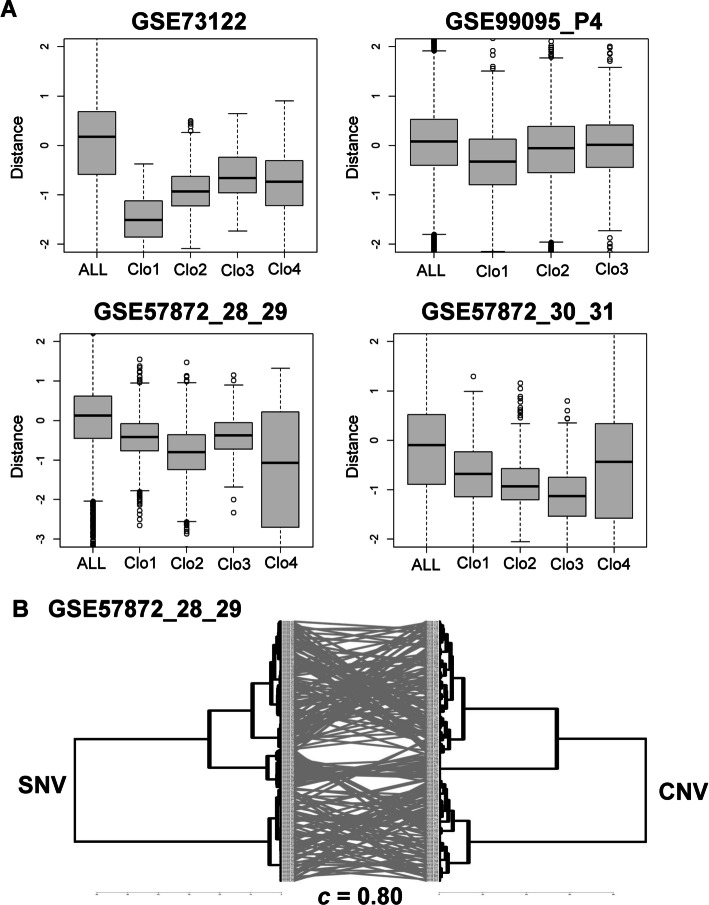
Concordance between clustering with SNV and CNV profiles. **A** Distances between cells were calculated by their CNV profiles and grouped by SNV profiles for datasets GSE73122, P4 of GSE99095, MGH28-29 of GSE57872, and MGH30-31 of GSE57872. Smaller distances within cells in the same SNV identified clone indicate that cells with the same mutation types share similar CNV profiles. Distances among all cells are larger than those among cells grouped by SNV profiles, which is consistent with the hypothesis that there is co-occurrence between SNVs and CNVs in some clones (Additional file 1: Fig. S1). **B** The tanglegram compares the phylogenetic clustering based on SNV and CNV profiles. Cells in clones defined by SNV also appear in the same clones defined by CNV (MGH28-29 of GSE57872). The cophenetic correlation between the two clustering results is 0.80

We compared CNV distances between cells in clones defined by SNVs. We found that the distances between cells in the same SNV-derived clones were smaller than the distances between all cells, indicating that the clones had similar CNV profiles (Fig. 2A).

Using the 4 real datasets, we found that when cells were ordered using clustering results from the SNV mutation data, there was a clear pattern of CNVs, indicating a co-occurrence of SNVs and CNVs (Additional file 2 and Additional file 3, Fig. 3C).

**Fig. 3:**
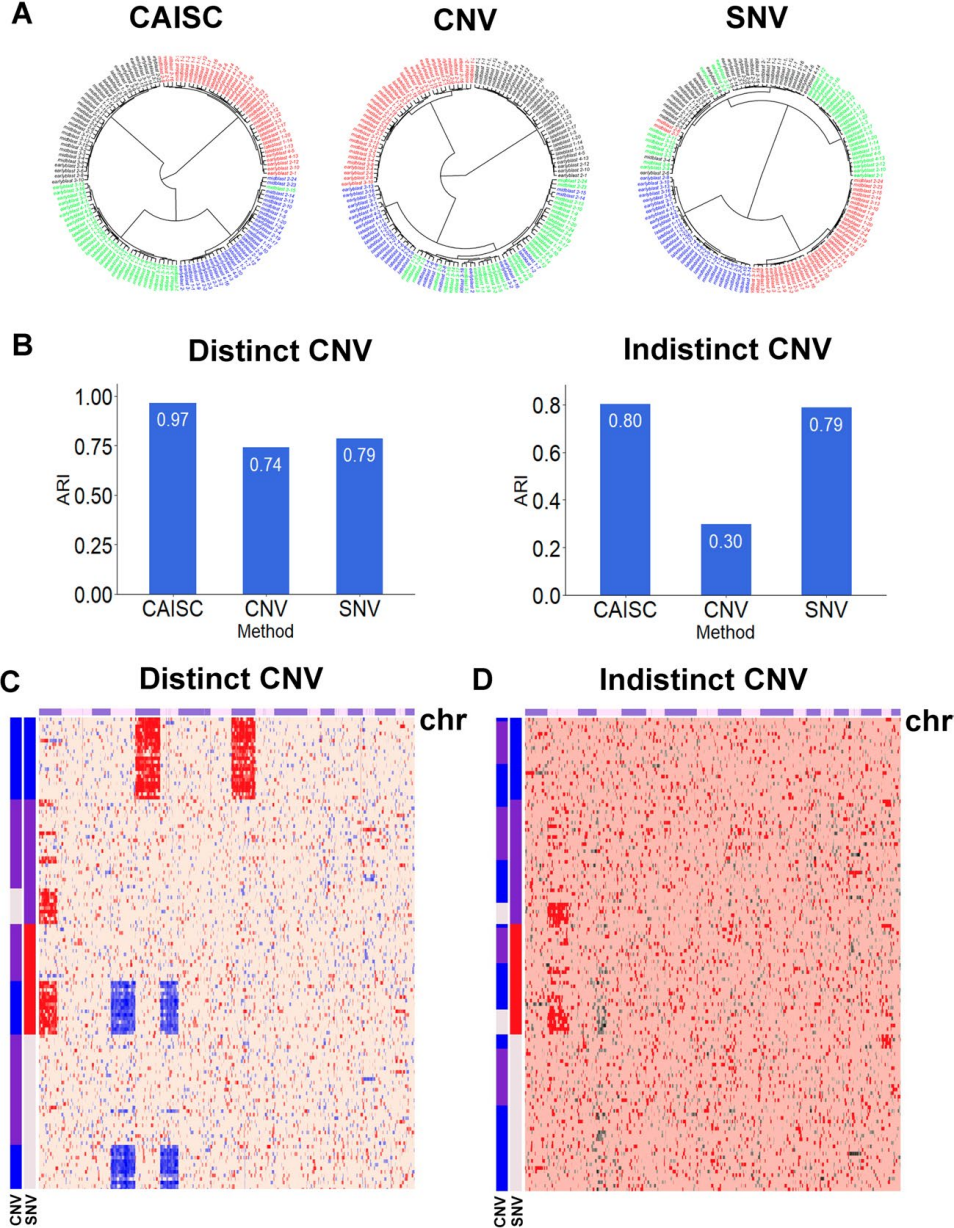
Accuracy of SNV, CNV, and CAISC clustering approaches on simulated data. **A** Fan dendrograms show simulated data with CNV, SNV and the CAISC clustering approaches, which agree with the predefined clone types colored with blue, purple, red, and lavender. **B** Adjusted Rand Index (ARI) was calculated for SNV, CNV, CAISC to evaluate effectiveness of clustering approaches with distinctly defined CNV and indistinct CNV. CNV heatmaps showing simulated data for two situations: with distinct and indistinct CNVs. These cells were clustered and ordered with SNV, then ordered with CNV, allowing for the identification of certain subclones. **C** When there are distinct CNV clones, with a large difference between subclones, patterns can be clearly identified. The red group in the SNV annotation can be further separated into two groups, each with different CNV. One group has chromosomal 1 loss and chromosomal 5,7 gains, while the other group has no obvious chromosomal alterations. **D** When there are indistinct CNV clones, with only slight differences between them, no clear patterns are apparent

**Fig. 4:**
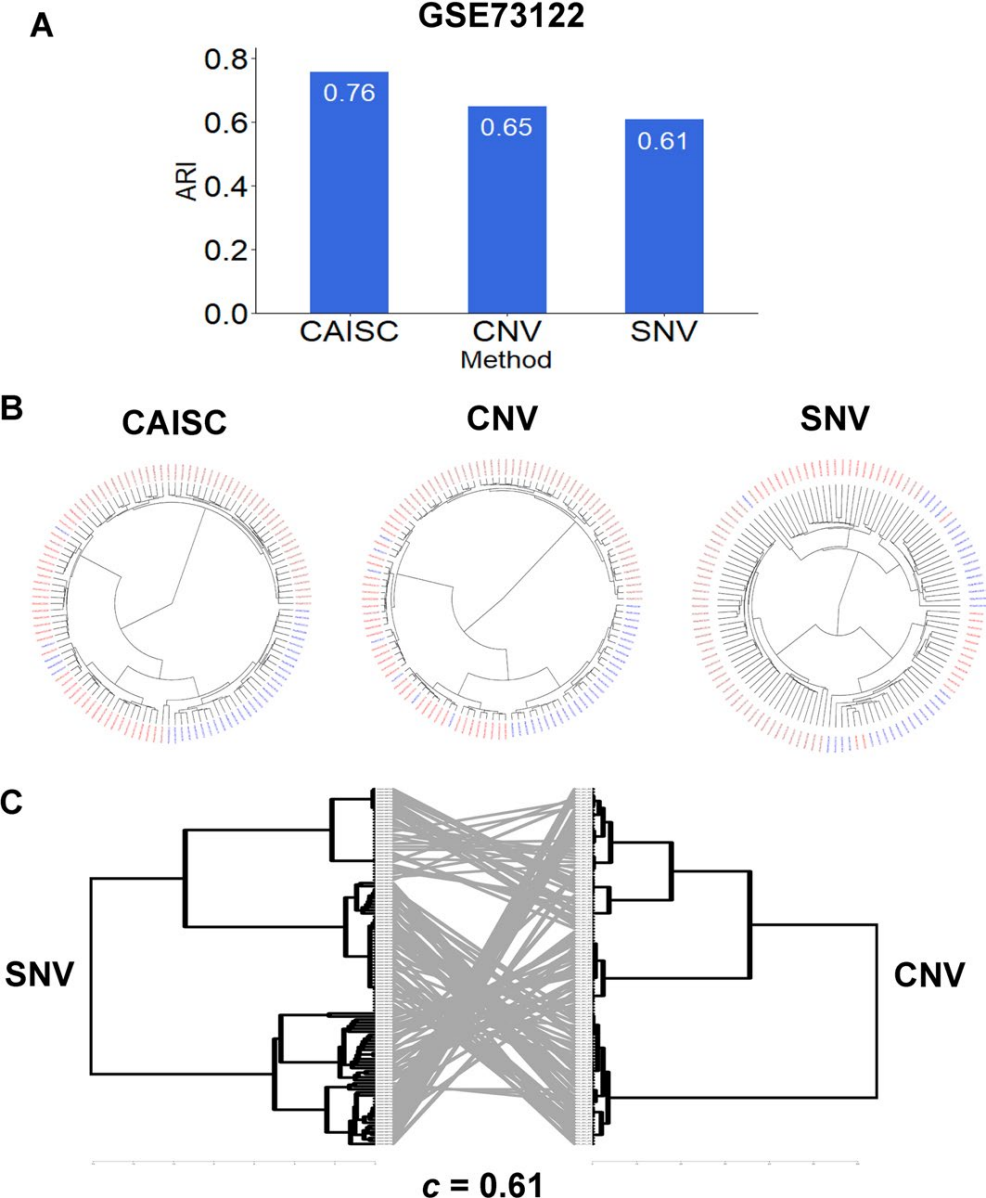
Evaluation of clustering accuracy using ARI on GSE73122 whose clone types are known. **A** ARI values show the integration performance of CAISC compared to CNV and SNV approaches. **B** Fan dendrograms showing the clustering results with SNV, CNV, and integrated approaches. **C** The tanglegram compares the phylogenetic clustering based on SNV and CNV profiles. Cells in clones defined by SNV also appear in the same clones defined by CNV. The cophenetic correlation between the two clustering results is 0.61

Finally, we assessed the concordance of results from scSNVs and scCNVs. Figure 2B shows the tanglegrams of the results. Cells that appear in the same clusters of scSNVs tended to also appear in the same clusters defined by scCNVs [15]. High cophenetic correlation (0.80) indicated good concordance between clustering results of SNV and CNV (Fig. 2B). High concordance (cophenetic correlation = 0.61) was also observed in GSE73122, in which all cells come from the same patient (Fig. 4C).

## Accuracy assessment and clone identification using simulated data

We assessed CAISC against SNV- and CNV-based clustering approaches using the simulated dataset generated from GSE45719. This dataset contained both SNVs and artificial CNVs that included chromosome gain and loss (Fig. 3C). We define the CAISC, SNV, and CNV approaches as methods which applied hierarchical clustering on the integrated distance matrix (D_combined_), SNV distance matrix (D_SNV_), and CNV distance matrix (D_CNV_), respectively.

In Fig. 3A, the results of the CNV, SNV and CAISC approaches were compared to pre-defined clone types using fan dendrograms, and a significantly higher accuracy of clone identification by CAISC was evident.

Based on ARI values, the CAISC approach performed better (ARI = 0.97) than the SNV (ARI = 0.79) and CNV (ARI = 0.74) approaches (Fig. 3B).

In Fig. 3C, the red group clearly had two CNV subclones from a chromosome 1p and half 1q loss, and chromosomes 5 and 7 gains, while the other cluster had no distinct CNVs.

With these data, we also simulated the different number of CNVs of different sizes (from 1/16 to 9/10 of chromosomes) in the pre-defined clones, CAISC was able to extract meaningful clustering results, which were consistent with predefined cell categories (average ARI ≈ 0.85). When there were distinct CNVs in pre-defined clones, clustering was significantly improved. The advantage of integration was not significant when there were no distinct CNVs across clones (Fig. 3D).

### The entropy based approach can lower the weight of CNV matrix when CNV alterations are not distinct

As seen in Fig. 3, we demonstrated that CNV data were most useful in clone identification when there were distinct CNV patterns in simulation. The entropy method is a commonly used weighting technique that measures value dispersion in decision making: as the degree of dispersion increases, the degree of differentiation also increases, allowing for more information to be derived. When elements in a distance matrix deviate from random distribution, the entropy is low and there is more information inside to be extracted, and thus a greater weight.

We simulated data both with and without distinct CNV profiles. When cells had clear clonal definition, the entropy of the distance matrix should be lower (Additional file 4: Fig. S4A), and the weight given during network integration would be higher. For the GSE45719 dataset, we ran simulations both with and without CNVs. As expected, entropy decreased when there were distinct CNV patterns in pre-defined clones (right bars of Additional file 4: Fig. S4B).

### Integration of CNV to SNV identifies more clones from real scRNA-seq data

**Fig. 5:**
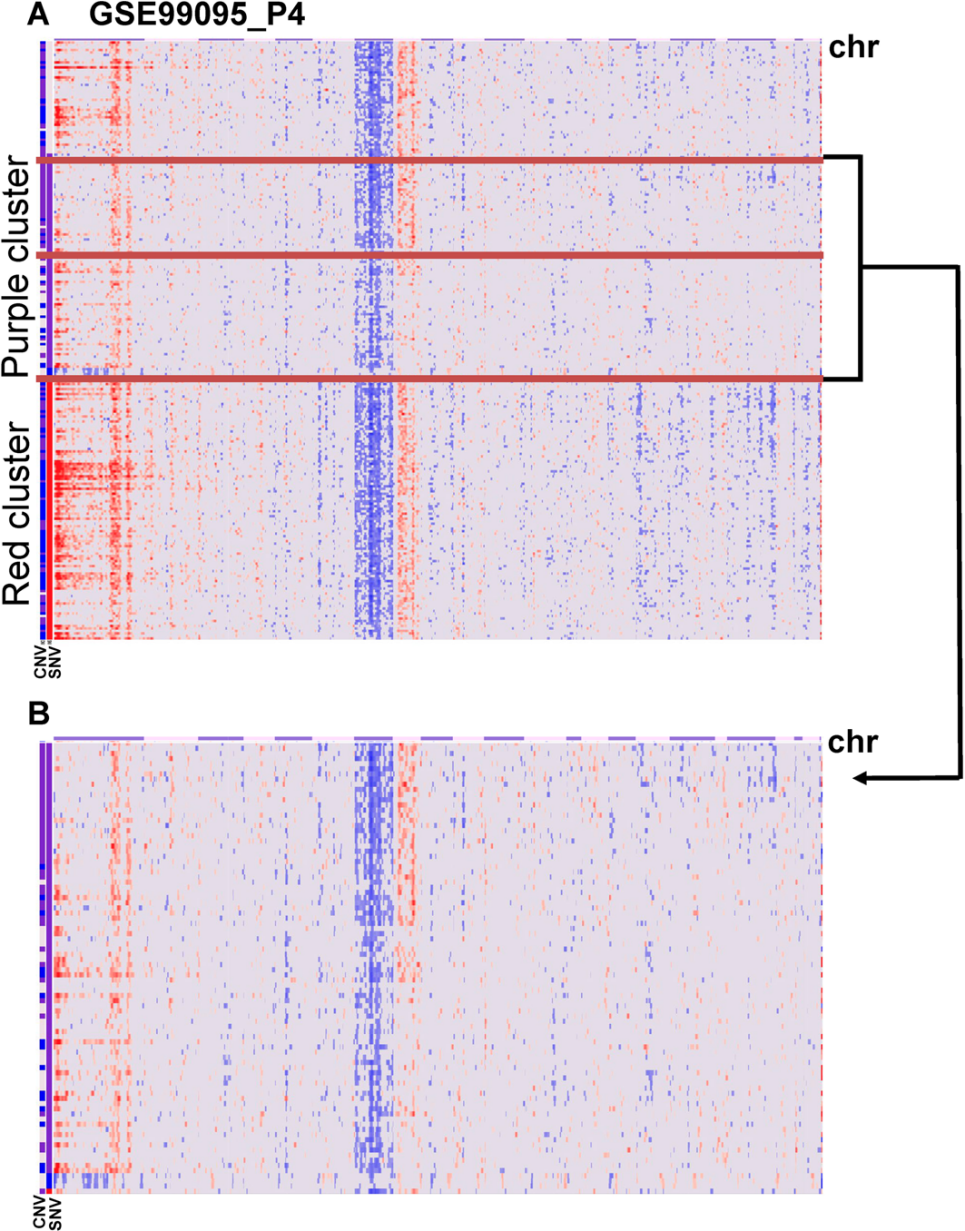
P4 of GSE99095 CNV heatmaps showing expression data generated by infercnv analysis. **A** These cells were clustered and ordered with SNV. The red lines show that there are 2 groups of CNV patterns in the purple group. The cells present in the purple group share the same SNV mutation profile. **B** The purple group in the SNV column can be further separated into three groups, each with different CNVs. One group has chromosome 8 gains, while the other group has no obvious chromosome 8 alterations. Both groups have 1q gain and chromosome 7 loss

We applied CAISC to analyze the scRNA-seq data of primary human glioblastomas (GSE57872) and bone marrow cells (P4 of GSE99095) [5, 6, 16]. CAISC generated CNV heatmaps of expression data of P4 in GSE99095 (Fig. 5). Clustering and ordering these cells with SNVs showed that a purple SNV cluster (row annotation on the left of Fig. 5A) could be further separated into two CNV subclones (Fig. 5B, highlighted by the horizontal red lines). One of these subclones had chromosome 8 gain, while the other had no obvious chromosomal alterations. Both had 1q gain and chromosome 7 loss. Clustering in this way allowed for examination of cells that had gained chromosome 8 and removed effects of SNV mutations and other CNVs on the transcriptome. CNV heatmaps of expression data of MGH30-31 of GSE57872 showed that a blue cluster originated from MGH31 (Additional file 2: Fig. S2). This cluster clearly had two CNV subclones, one with multiple chromosome gains and the other with indistinct chromosome gains. Both clusters had chromosome gain and chromosome loss. CNV heatmaps of expression data of MGH28-29 of GSE57872 showed that the lavender cluster comes only from MGH29, and it has two distinct CNV subclones (Additional file 3: Fig. S3). One had multiple chromosome gains, while the other had no distinct chromosome gain. Both had chromosome gains and chromosome losses. Thus, integrating CNV and SNV elucidated more clones from real scRNA-seq data that would have otherwise not been observed by clustering based only on SNV. The CAISC method allowed for identification of more subclone characteristics, as could be seen from identification of chromosome gains and losses as described above. One exception was that integration with CNV did not identify new subclones in GSE73122 data (Additional file 5: Fig. S5), which might indicate that no new clones had arisen from CNV (Additional file 1: Fig. S1).

### Accuracy assessment using real datasets

We benchmarked CAISC with two real datasets. First, we applied CAISC to a renal cell carcinoma dataset (GSE73122). This dataset contained 116 cells obtained from three tumors from one patient [17]. The three tumor types were patient-derived xenograft (PDX), metastasis to the lung (Pt_mRCC), and a PDX of the lung metastasis renal cell carcinoma (PDX_mRCC). Cells should share common early driver mutations, but the metastasis and in vitro culture should have generated new SNVs and CNVs. Thus, the three tumors should be clonally distinct. All three algorithms could distinguish cells with high accuracy (Fig. 4A, B). Based on ARI values, the CAISC approach performed better (ARI = 0.76), than the SNV (ARI = 0.61) and CNV (ARI = 0.65) approaches did (Fig. 4A, B). Furthermore, there was high consistency between results for scSNVs and scCNVs (Fig. 4C).

Second, we applied CAISC to analysis of data from a study of primary human glioblastomas (GSE57872_28_29, GSE57872_30_31 datasets). The patients in these datasets share many SNVs and CNVs because there were many common driver mutations. We first assessed whether CAISC separated MGH28 cells from MGH29 cells and MGH30 cells from MGH31 cells, since inter-individual similarity of SNVs and CNVs should far exceed intra-individual similarities. All three approaches could separate cells with high accuracy (Additional file 6: Fig. S6A). For GSE57872_28_29, CAISC again performed better (ARI = 0.64), than the SNV (ARI = 0.51) and CNV (ARI = 0.62) approaches did (Additional file 6: Fig. S6A). For MGH30 and MGH31 of GSE57872, we found that integration did not increase accuracy, but CAISC was able to identify more subclones (Additional file 2 and Additional file 6: Fig. S6B).

In summary, SNV-based clustering approaches were not able to identify certain clones that occurred as a result of distinct CNV (Additional file 1: Fig. S1), thus leading to biased results. The above evaluations of CAISC compared to the SNV and CNV methods showed that CAISC (the integrated approach) could identify more subclones, increased the accuracy of subclone identification, and revealed details about clones in real data.

### Integration of SNV and CNV allows more accurate examination of expression changes between subclones

In the comparison of two groups, matching cofactors is required to improve study efficiency [18]. Detailed identification of subclones allows for removal of co-factor effects. In our previous study, P4 was excluded from our analysis due to its complicated pattern of mutation variants [6]. When focusing on the purple cluster defined by SNV (Fig. 5B), effects of single nucleotide mutations and other CNVs would be removed because all cells in the purple cluster had the same mutation profile, and monosomy 7 and 1q duplication. Differentially expressed (DE) genes were generated by comparing trisomy 8 cells and non-trisomy 8 cells in the purple cluster. DE genes of trisomy 8 cells were associated with the TNF-α related pathways, with annotation of GO:0071706 (tumor necrosis factor superfamily cytokine production) [19]. TNF-α plays important roles in the pathophysiology of MDS by inhibiting normal hematopoiesis and inducing programmed cell death of normal CD34 + cells [20]. Patients with MDS involution and trisomy 8 can be successfully treated with adalimumab, a TNF blocker. DE genes are also implicated in apoptosis, with annotation of GO:0007254 (JNK cascade) and GO:0032872 (regulation of stress-activated MAPK cascade). Our analysis is preliminary due to the limited number of cells from a single patient, but it implies the potential of CAISC to examine expression alterations despite extensive genetic complexity in tumor cells.

### Interactive examination of identified clones with the Shiny app

In addition to generating heatmap figures, CAISC allows us to use the Shiny web app to visualize results and interactively examine identified clones, using a third-party interactive heatmap package (https://github.com/jokergoo/InteractiveComplexHeatmap). This package allows for data examination and visualization, through zooming and focusing on selected clones and chromosome regions (Additional file 7: Fig. S7). The left panel shows an original heatmap of all cells and all chromosomes. Parts of cells and chromosomal regions can be shown in the right panel when the rectangular selection is indicated. Though this is one way to visualize results, users can choose to use other methods at their own discretion.

## Discussion

Incorporating CNVs into clone definition and reconstruction of tumor phylogeny should be helpful in elucidating tumor progression, as CNVs are frequent with tumor hypermutability. Further integration of both types of data is necessary, considering their co-occurrence in cellular clones and the high background noise inherent in single cell methods to detect CNV and SNV [1, 21, 22]. Our method is not applicable to the data from the currently popular platform of 10 × Genomics, in which only the 5’ or 3’ end of the mRNA is sequenced. Measuring only small regions of the mRNA leads to an insufficient number of SNVs for individual cells, which would not be ideal for BAM files by Cellranger.

SNV based clustering approaches are unable to identify clones that occur as a result of CNV, thus leading to incomplete results. CAISC uses an integrated entropy weighted strategy to combine SNV and CNV results from DENDRO and infercnv respectively, for the purpose of obtaining more accurate and robust clustering results. There is no perfect algorithm to call SNV [23]. In this study, we used the GATK best practices pipeline to call the SNV [24], which is computationally complex and usually relies on resources from a high performance computing (HPC) cluster. We have included a script in our GitHub repository for running GATK on a HPC cluster. It takes about 5 h to finish calling SNVs in an HPC cluster with about 50 CPUs and 50 GB RAM.

The Pearson correlation coefficient of CNV profiles is used to calculate the distance between cells. However, we found that Pearson correlation was not sensitive when there were only small CNVs in clones. Furthermore CAISC did not always perform better than the approach with SNVs and CNVs only, meaning there is room to improve our algorithm, Pearson correlation worked well when there were large CNVs, such as chromosomal or sub-chromosomal aneuploidy. Other indices, such as mutual information and partial correlation, as well as the filtering strategies to only keep informative CNVs, will be examined in the future. In our current version of CAISC, we used hierarchical clustering, which involved many arbitrary decisions, such as single linkage, complete linkage, centroid linkage, or full linkage clustering. We will examine other advanced clustering approaches, such as density-based spatial clustering of applications with noise (DBSCAN) algorithms, which can detect arbitrarily-shaped clusters [25]. Our software focuses on scRNA-seq data only, but our methods can be applied to the scDNA-seq and scRNA-seq data of the same cells [26]. Our current analysis was based on the SNVs in transcriptome regions only, and we expect to have better performance when there is a dataset which separates and sequences genomic DNA and full-length mRNA from the same single cells. This is because high number of SNVs in non-transcriptional regions provide more information, and CNVs from DNA and mRNA can be validated against each other [27, 28].

## Conclusions

As the technology of variant calls develops, it becomes more important to model different types of available signals mathematically in order to fully characterize tumor evolution. In the present study, we included SNVs, which are point mutations, and CNVs, which are larger structural variations. There are multiple levels of genetic heterogeneity associated with cancer, including single nucleotide polymorphisms, microsatellite shifts, copy number variations, and karyotypic variations (structural aberrations and aneuploidy). Thus, it is necessary to integrate multiple levels of genetic variations when studying tumor heterogeneity. Our entropy-based framework is suitable for integration of multiple types of information. Evaluations of CAISC compared to other methods showed that the integrated approach could increase accuracy of subclone identification, characterize frequency and mutation profiles of clones, and infer phylogenetic relationships among clones from real data.

## Method

### Cell–cell distance matrix construction using SNV and CNV profiles by DENDRO and infercnv

DENDRO (DNA based EvolutioNary tree preDiction by scRNA-seq technOlogy) is a statistical and computational framework that creates a phylogenetic tree of tumor subclones based on genetic divergence, which is calculated from cell–cell DNA mutations detected in scRNA-seq [8]. The framework factors in technical dropout, expression stochasticity, and sequencing errors. First, raw scRNA-seq data was aligned with the STAR 2-pass method which is commonly used to call CNV and SNV from scRNA-seq data [29]. Next, the resulting BAM files entered a pipeline of processing steps, starting from sorting, to joining read groups, removing duplicated reads, removing overhangs into intronic regions, realigning, and finally recalibration. The GATK tool, Haplotype Caller, was used to call variants in these processed BAM files to generate VCF files, which were subsequently filtered so that only mutations that occurred in a minimal set number of cells were retained [23, 24]. Subsequently, alternative allele read counts, total allele read counts, and a mutation profile matrix for each cell and loci were extracted from the filtered VCF files. These data were filtered to remove low-expressed and high-dropout-rate cells and to calculate cell–cell genetic divergence based on total reads and mutation frequencies. The results of these calculations were used to create a cell–cell distance matrix of mutations [8].

Infercnv is a computational tool used to analyze tumor scRNA-seq data: it identifies somatic large-scale chromosomal copy number variations (gains or deletions of chromosomes) [7, 16]. Infercnv analyzes the expression intensity of genes across the tumor genome and compares them to that of a set of “normal” reference cells. In order to generate an infercnv object, three inputs are required. First, a raw counts matrix containing assigned read counts must be generated, in which rows are genes, and columns are cells. Second, a sample annotation file was used to define different cell categories and direct how cells should be grouped. Third, a gene ordering file provided a chromosomal location for each gene. Once the infercnv object was created, the expression data was used to compute correlation values. Correlations allowed the computation of distance values, from which a normalized cell–cell distance matrix of mutations could be generated. The distance matrix was used for hierarchical clustering to define CNV clones and examine intratumor heterogeneity [4].

The ability of a CNV to differentiate between clone clusters depends on its characteristics. In this study, “distinct” CNV was defined as a CNV which had (1) highly heterogeneous profiles across different clones and showed homology in the same clone; and (2) a strong signal, which was high enough to be detected from the noisy scRNA-seq data. A CNV with a weak signal or a subtle difference between clones is considered “an indistinct CNV”.

Using DENDRO and infercnv, we generated two cell–cell distance matrices for integrative analysis:

1 $$D_{SNV} = \left[ {\begin{array}{*{20}c} {D_{SNV}^{{\left( {c_{1} ,c_{1} } \right)}} } & \cdots & {D_{SNV}^{{\left( {c_{1} ,c_{N} } \right)}} } \\ \vdots & \ddots & \vdots \\ {D_{SNV}^{{\left( {c_{N} ,c_{1} } \right)}} } & \cdots & {D_{SNV}^{{\left( {c_{N} ,c_{N} } \right)}} } \\ \end{array} } \right]\quad D_{CNV} = \left[ {\begin{array}{*{20}c} {D_{CNV}^{{\left( {c_{1} ,c_{1} } \right)}} } & \cdots & {D_{CNV}^{{\left( {c_{1} ,c_{N} } \right)}} } \\ \vdots & \ddots & \vdots \\ {D_{CNV}^{{\left( {c_{N} ,c_{1} } \right)}} } & \cdots & {D_{CNV}^{{\left( {c_{N} ,c_{N} } \right)}} } \\ \end{array} } \right]$$

### Integration of SNV and CNV matrices with an entropy weighted method

The computational framework of CAISC is shown in Fig. 1, in which we used an entropy weighted method for integration.

The matrices derived from DENDRO and infercnv can be regarded as a weighted network, in which each cell is a node, and the distance between nodes are edges. Entropy measures the structural complexity of a network, thus its concept can be utilized to integrate multiple weighted graphs or networks, or in this case, to integrate the cell–cell distance matrices generated by the DENDRO and infercnv analyses. For each edge in the intersection of the edge sets of the two matrices in Eq. 1, a new edge weight is calculated based on the edge weights of the two networks [30–32] to generate an integrated matrix.

For a given graph G with vertex $v_{i}$, let $d_{i}$ be the degree of $v_{i}$. For an edge $v_{i} v_{j}$, we defined:

2 $$p_{ij} = \frac{{w\left( {v_{i} v_{j} } \right)}}{{\mathop \sum \nolimits_{j = 1}^{{d_{i} }} w\left( {v_{i} v_{j} } \right)}}$$

where $w(v_{i} v_{j} )$ is the weight of the edge $v_{i} v_{j}$ and $w(v_{i} v_{j} ) > 0$. In our case, the weight was the normalized distance $D_{i,j}$ between cells i and j. The node entropy H for a network *k* was defined by $p_{ij}$ in Eq. 2:

3 $$H\left( k \right) = - \mathop \sum \limits_{j = 1}^{{d_{i} }} p_{ij} {\text{log}}\left( {p_{ij} } \right)$$

We then calculated an $\alpha$ value for each network *k* of two networks with Eqs. 4 and 5. The integration parameters $\alpha_{k}$ are inferred by normalizing $C_{k}$. A smaller value of $\alpha_{k}$ should be given to the network with larger entropy, as a network with large entropy is high in disorderly structural diversity. A weight function which decreases with the increase of H is defined as follows, same as in [33]:

4 $$C_{k} = 1 - e^{{ - \frac{1}{{\left[ {H\left( k \right)} \right]^{\Theta } }}}}$$

where θ > 0 is an adjustment parameter, which can be properly selected by network property [33]. We assigned θ = 2 because we were integrating two networks. Given our two distance matrices, we calculated two C values: $C_{CNV}$ and $C_{SNV}$ with Eq. 4, generated from the infercnv and DENDRO data, respectively. Our function was specifically designed to restrict the integration parameter $C_{k}$ in the area (0, 1), with a sum of 1. We designed the integration parameters $\alpha_{k}$ by normalizing $C_{k}$ as follows:

5 $$\alpha_{CNV} = \frac{{C_{CNV} }}{{C_{CNV} + C_{SNV} }}\quad \alpha_{SNV} = \frac{{C_{SNV} }}{{C_{CNV} + C_{SNV} }}$$

Using the alpha values in Eq. 5, we could compute a new integrated matrix $D_{combined}$ from matrices $D_{CNV}$ and $D_{SNV}$ in Eq. 1:

6 $$D_{combined} = \alpha_{CNV} D_{CNV} + \alpha_{SNV} D_{SNV}$$

### The elbow point and gap statistic for estimating the optimal number of clusters

One challenge in cluster analysis is estimating the optimal number of clusters. We included two approaches in the CAISC package for users to select: elbow point and gap statistic [34].

A common method used to determine this estimate is the elbow point, in which the error measure $W_{k}$ (within cluster dispersion) is plotted against the number of clusters.

Let $D_{r}$ represent the sum of all intra-cluster distances between points ($x_{i} , x_{j}$) in a given cluster $C_{r}$ containing $n_{r}$ points, calculated using the squared Euclidean distance.

$W_{k}$ is calculated by summing the normalized $D_{r}$ in order to determine the pooled within-cluster sum of squares around the cluster means.

$$\begin{aligned} D_{r} & = \mathop \sum \limits_{{x_{i} \in C_{r} }} \mathop \sum \limits_{{x_{j} \in C_{r} }} \left| {\left| {x_{i} - x_{j} } \right|} \right|^{2} \\ W_{k} & = \mathop \sum \limits_{r = 1}^{k} \frac{1}{{2n_{r} }}D_{r} \\ \end{aligned}$$

While initially, the error measure decreases monotonically as the number of clusters *k* increases, eventually at some *k* onwards, the decrease flattens. This point is known as the “elbow” and can be used to estimate the optimal number of clusters. However, the elbow point cannot always be definitively identified.

Alternatively, the gap statistic method can be used to formalize the elbow heuristic [34]. The graph of ${\text{log}}\left( {W_{k} } \right)$ can be standardized by comparison with its expectation under an appropriate null reference distribution of the data, and the optimal number of clusters can be estimated as the value of *k* for which ${\text{log}}\left( {W_{k} } \right)$ falls the farthest below this reference curve.

### Construction of simulated data

We generated simulation data by adopting an scRNA-seq dataset of GSE45719 as the reference [35]. The clonal definition of each cell within this dataset was well defined. For every simulated locus, we sampled an SNP from this reference, same as in a study from DENDRO [8]. We randomly assigned one allele of the sampled SNP as the mutated allele for cells with mutations. When cells lacked mutation values, we set the mutated allele counts as 0 and used the sum of the two alleles from the reference as the total read counts. To simulate random sequencing errors, binomial noise was also added to read counts [8]. Simultaneously, we downloaded the expression data and created artificial CNVs in different pre-defined clones. The simulations included both chromosome or sub-chromosome gain (with 50% or 100% increase of expression) and chromosome or sub-chromosome loss (with 50% decrease of expression). The script for one simulation is available at https://github.com/lizamathews/CAISC.

### Real datasets for assessment

The scRNA-seq datasets of three studies: GSE99095, GSE73122, and GSE57872 from GEO (https://www.ncbi.nlm.nih.gov/geo/) were used to examine the performance of CAISC. The first dataset (GSE99095) was obtained from human bone marrow cells, 391 control cells from 4 healthy donors and 588 cells from 5 patients with bone marrow failure and cytogenetic abnormalities [6]. Previously, we were able to identify CNVs within this dataset, but did not identify SNVs due to low sequencing read numbers and limited sequencing coverage [6]. A renal cell carcinoma dataset of GSE73122 included three types of known clones to benchmark accuracy [17]. We also applied our approach on primary human glioblastoma data (GSE57872) to examine clones and genetic heterogeneity. We combined MGH28 and MGH29, and MGH30 and MGH31 for data analysis to examine whether CAISC could separate cells from different patients [4, 36]. (The same assessment strategy (i.e. accuracy of separating cells from patients) has been used by others [8].)

### Evaluation of CAISC performances

We compared the performance of CAISC to SNV-only and CNV-only clustering approaches using adjusted Rand Index (ARI). This index evaluates the capability of an algorithm to separate elements belonging to different classes. It analyzes each pair of elements and not only evaluates the separation of elements in different groups, but also the relation of elements in the same group. ARI is a more sensitive performance index than the Rand Index, can accept constant values like 0, and assumes the partitions are chosen at random [1, 8].

$$ARI = \frac{{\left( {\begin{array}{*{20}c} n \\ 2 \\ \end{array} } \right)\left( {a + d} \right) - \left[ {\left( {a + b} \right)\left( {a + c} \right) + \left( {c + d} \right)\left( {b + d} \right)} \right]}}{{\left( {\begin{array}{*{20}c} n \\ 2 \\ \end{array} } \right)^{2} - \left[ {\left( {a + b} \right)\left( {a + c} \right) + \left( {c + d} \right)\left( {b + d} \right)} \right]}}$$

**Table:** 

*Partition*	*V*	
*U*	# of Pairs in same group	# of Pairs in different groups
# of Pairs in same group	*a*	*B*
# of Pairs in different groups	*c*	*D*

The expected value is 0 for random partitioning, and the maximum value is 1 for perfect agreement between the two partitions. This index was used to evaluate how well our combined algorithm clustered cells compared to SNV and CNV clustering approaches.

## Availability and requirements

Project name: CAISC home page: https://github.com/lizamathews/CAISC. Operating system(s): Cross-platform. Programming language: Shell, R License: GPL-3.0.

## Supplementary Information

**Additional file 1: Figure S1**. Integrating SNV and CNV data identifies subclones that would have otherwise been missed with only SNV data. A) SNV and CNV subclone matrices show mutations in different samples and subclones. Rows represent samples. Columns represent subclones. B) Phylogeny tree generated with only SNV data C) Phylogeny tree generated with both SNV and CNV data. When analyzing data with SNV only, clones A and C and clones B and E were not identified as distinct subclones.

**Additional file 2: Figure S2**. MGH 30-31 of GSE57872 CNV heatmaps calculated with Infercnv analysis. A) The cells were clustered and ordered with SNV. (B) The blue group in SNV can be further separated into two groups with different CNVs. Though there are many of the same CNV alterations (6 gain, 2p gain), one group has chromosome 10 and 14 loss. The other group has no obvious alterations on these two chromosomes.

**Additional file 3: Figure S3**. MGH 28-29 of GSE57872 CNV heatmaps calculated with infercnv analysis. A) The cells were clustered and ordered with SNV. (B) The lavender group in SNV can be further separated into two groups with different CNVs. One group has chromosome 10 and 15 losses. Another group has no obvious alterations on these two chromosomes.

**Additional file 4: Figure S4**. Entropy of distance matrices with and without subclones. A) Each node is a cell, and the edge widths represent the proximity (reciprocal of distances) between cells. Compared to cells that belong to different clones, cells that are in the same clones have a shorter distance between them. The entropy of a distance matrix with subclones will be lower, and therefore a higher weight will be given for integration. B) Entropy of simulated distance matrix calculated by the gene expression with subclones (6–11) and without subclones (1–5).

**Additional file 5: Figure S5**. GSE73122 CNV heatmaps calculated with infercnv analysis. The cells were clustered and ordered with SNV. No new subclones were found after integration with CNVs.

**Additional file 6: Figure S6**. Evaluation of MGH28-29 of GSE57872 and MGH30-31 of GSE57872 datasets with SNV, CNV, and CAISC. A) Fan dendrograms show MGH28-29 of GSE57872 data with SNV, CNV and the CAISC clustering approaches. B) Fan dendrograms show MGH30-31 of GSE57872 data with SNV, CNV and the CAISC clustering approaches.

**Additional file 7: Figure S7**. The screen shot of interactively examination of the identified clones with Shiny app.

## Data Availability

CAISC is an open-source R package available at https://github.com/lizamathews/CAISC with license GPL-3.0. Public datasets for simulation analysis and accuracy validation using bone marrow cell, renal cell carcinoma, primary human glioblastomas, and breast cancer cell data can be found at the National Center for Biotechnology Information Gene Expression Omnibus (GEO) under accession numbers GSE45719, GSE99095, GSE73122, GSE57872, and GSE75688 respectively.

## Abbreviations

CNV: Copy number variation
SNV: Single nucleotide variation
scRNA-seq: Single-cell RNA sequencing
ARI: Adjusted rand index
CAISC: Clonal architecture with integration of SNV and CNV
