# Supplementary figures and images for: CAISC: A software to integrate copy number variations and single nucleotide mutations for genetic heterogeneity profiling and subclone detection by single-cell RNA sequencing
Source: BMC Bioinformatics. 2022 Mar 21;23(Suppl 3):98. doi: 10.1186/s12859-022-04625-x (PMC8939069; doi:10.1186/s12859-022-04625-x)

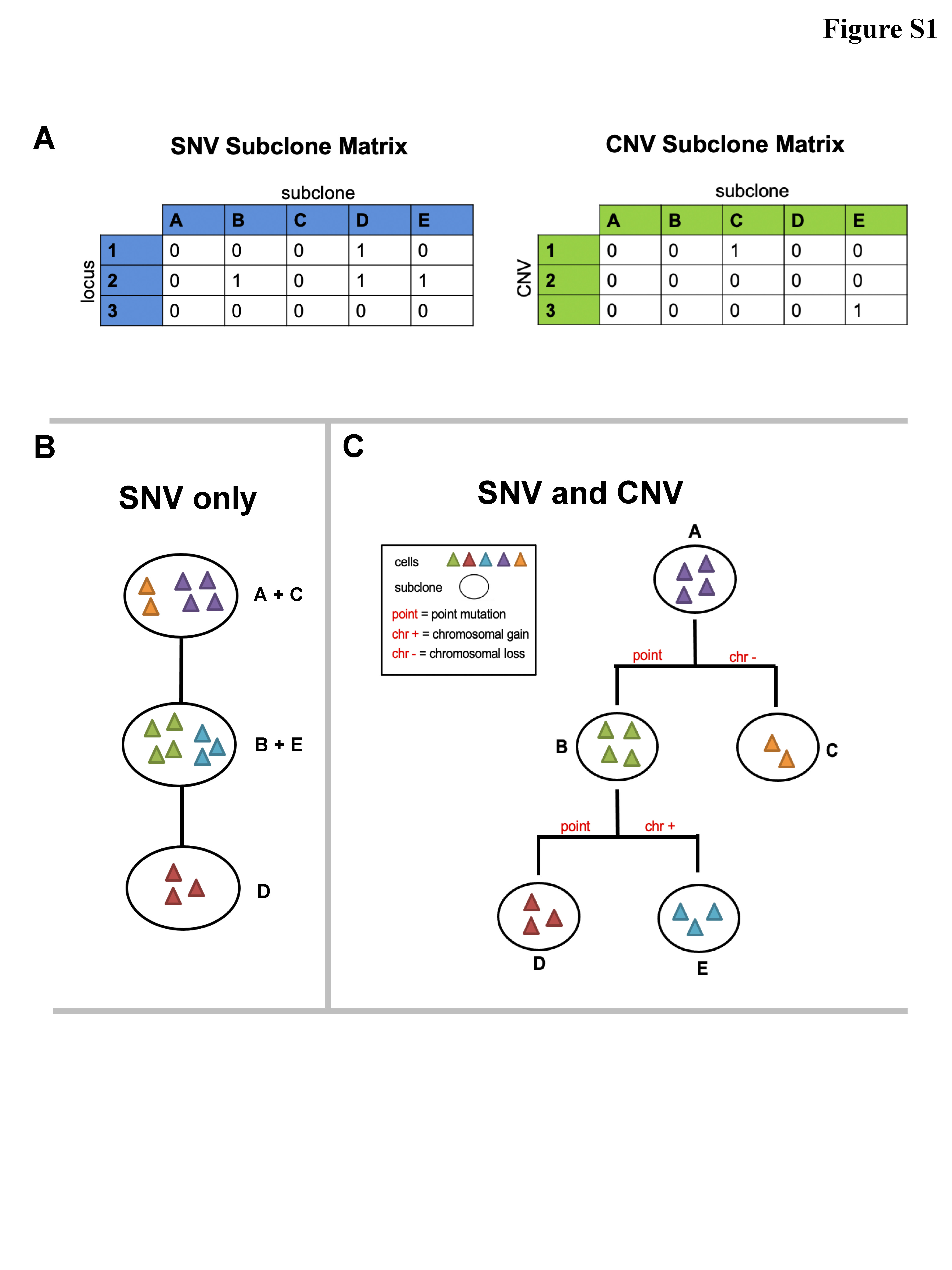

Supplement: Supplementary file 1 — Additional file 1: Figure S1. Integrating SNV and CNV data identifies subclones that would have otherwise been missed with only SNV data. A) SNV and CNV subclone matrices show mutations in different samples and subclones. Rows represent samples. Columns represent subclones. B) Phylogeny tree generated with only SNV data C) Phylogeny tree generated with both SNV and CNV data. When analyzing data with SNV only, clones A and C and clones B and E were not identified as distinct subclones. [file 12859_2022_4625_MOESM1_ESM.jpg]

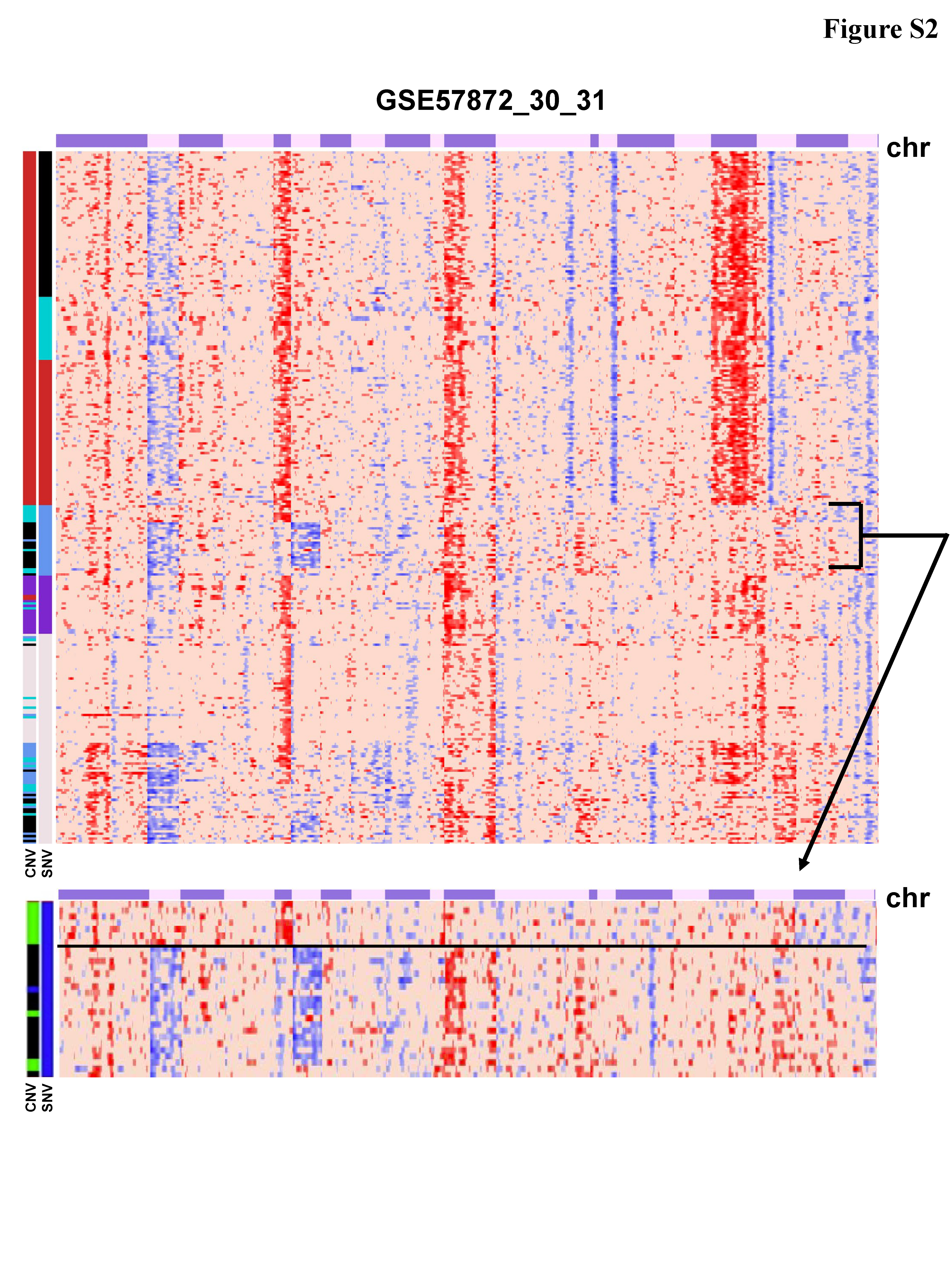

Supplement: Supplementary file 2 — Additional file 2: Figure S2. MGH 30-31 of GSE57872 CNV heatmaps calculated with Infercnv analysis. A) The cells were clustered and ordered with SNV. (B) The blue group in SNV can be further separated into two groups with different CNVs. Though there are many of the same CNV alterations (6 gain, 2p gain), one group has chromosome 10 and 14 loss. The other group has no obvious alterations on these two chromosomes. [file 12859_2022_4625_MOESM2_ESM.jpg]

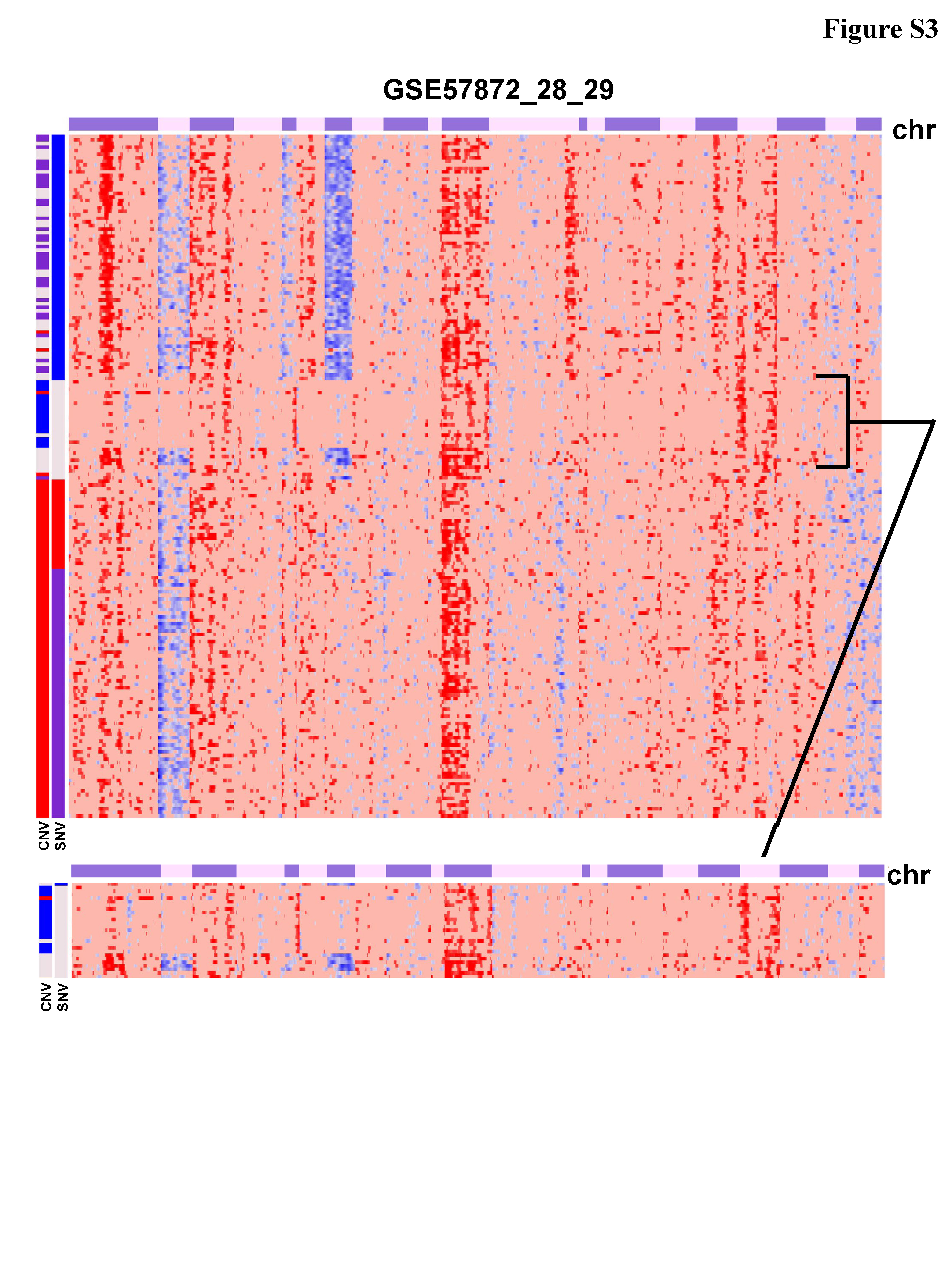

Supplement: Supplementary file 3 — Additional file 3: Figure S3. MGH 28-29 of GSE57872 CNV heatmaps calculated with infercnv analysis. A) The cells were clustered and ordered with SNV. (B) The lavender group in SNV can be further separated into two groups with different CNVs. One group has chromosome 10 and 15 losses. Another group has no obvious alterations on these two chromosomes. [file 12859_2022_4625_MOESM3_ESM.jpg]

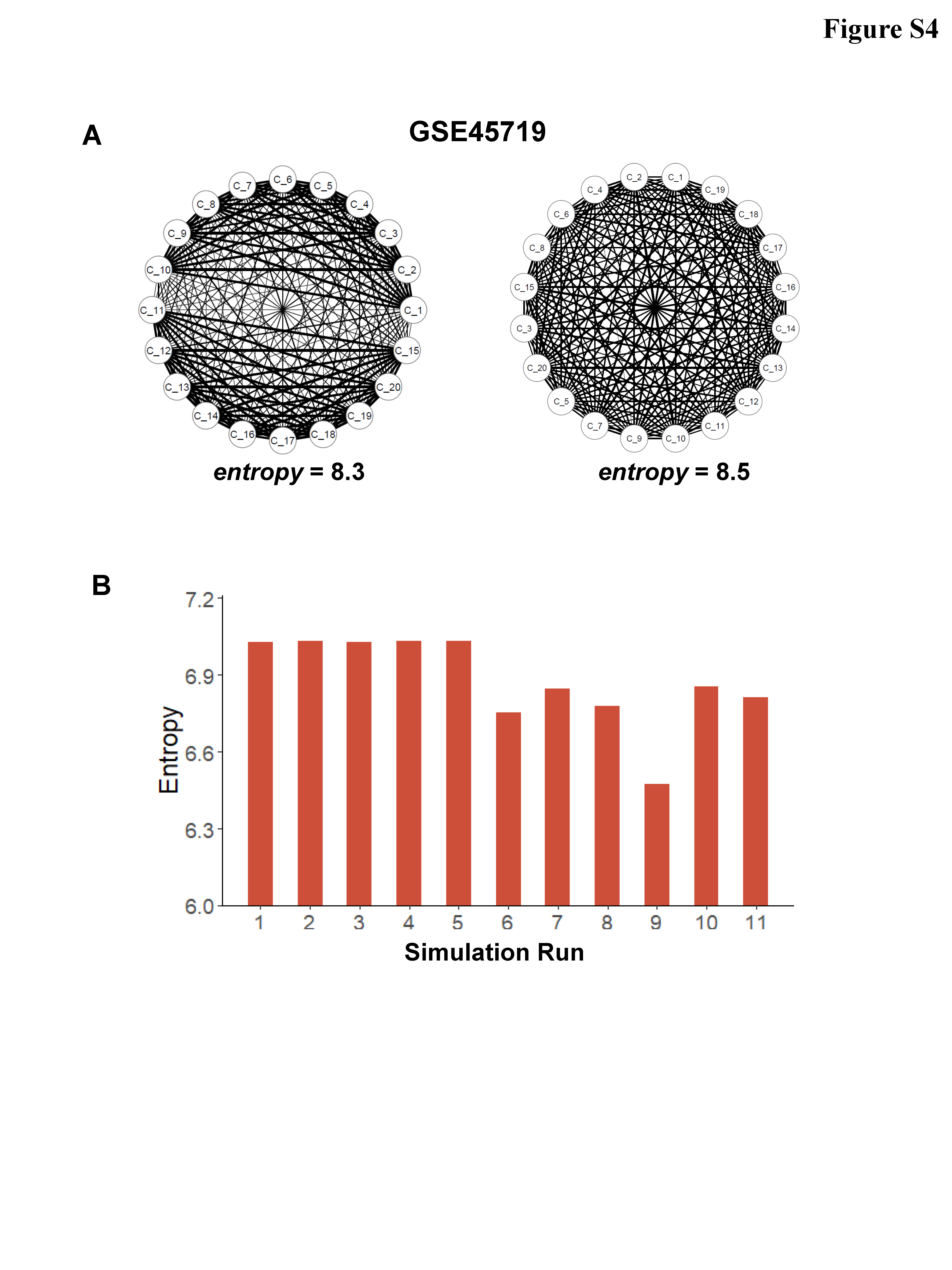

Supplement: Supplementary file 4 — Additional file 4: Figure S4. Entropy of distance matrices with and without subclones. A) Each node is a cell, and the edge widths represent the proximity (reciprocal of distances) between cells. Compared to cells that belong to different clones, cells that are in the same clones have a shorter distance between them. The entropy of a distance matrix with subclones will be lower, and therefore a higher weight will be given for integration. B) Entropy of simulated distance matrix calculated by the gene expression with subclones (6–11) and without subclones (1–5). [file 12859_2022_4625_MOESM4_ESM.jpg]

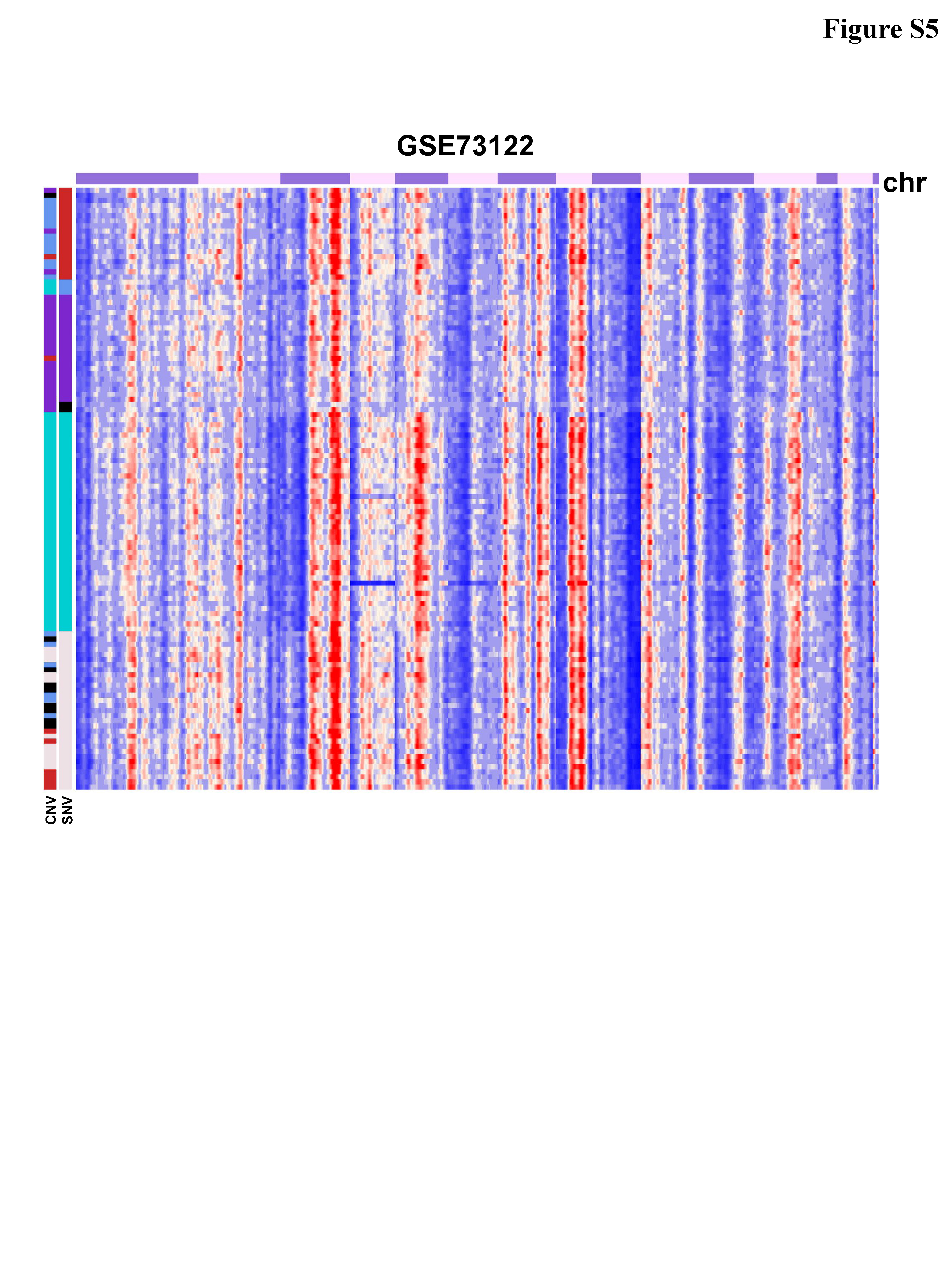

Supplement: Supplementary file 5 — Additional file 5: Figure S5. GSE73122 CNV heatmaps calculated with infercnv analysis. The cells were clustered and ordered with SNV. No new subclones were found after integration with CNVs. [file 12859_2022_4625_MOESM5_ESM.jpg]

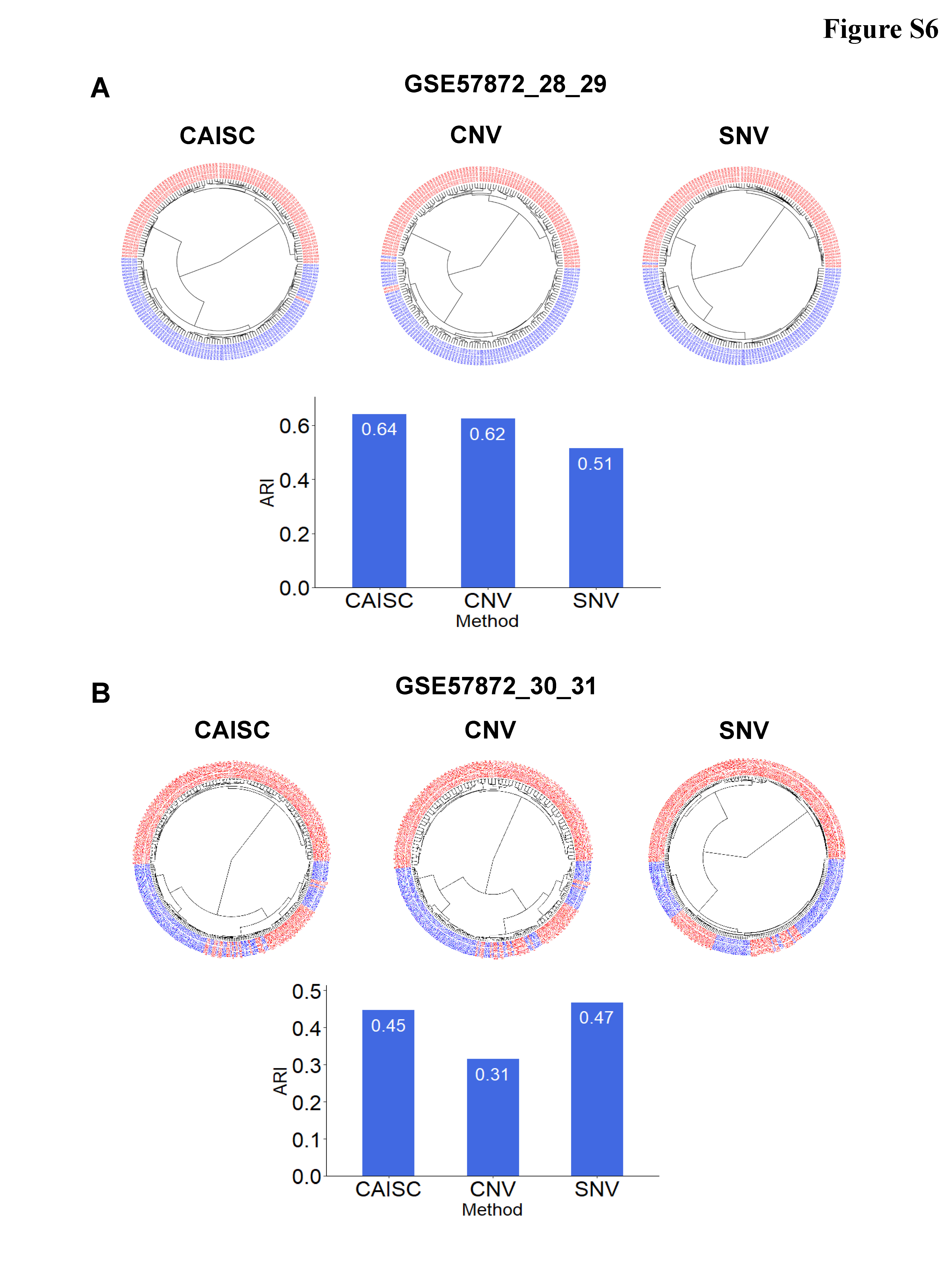

Supplement: Supplementary file 6 — Additional file 6: Figure S6. Evaluation of MGH28-29 of GSE57872 and MGH30-31 of GSE57872 datasets with SNV, CNV, and CAISC. A) Fan dendrograms show MGH28-29 of GSE57872 data with SNV, CNV and the CAISC clustering approaches. B) Fan dendrograms show MGH30-31 of GSE57872 data with SNV, CNV and the CAISC clustering approaches. [file 12859_2022_4625_MOESM6_ESM.jpg]

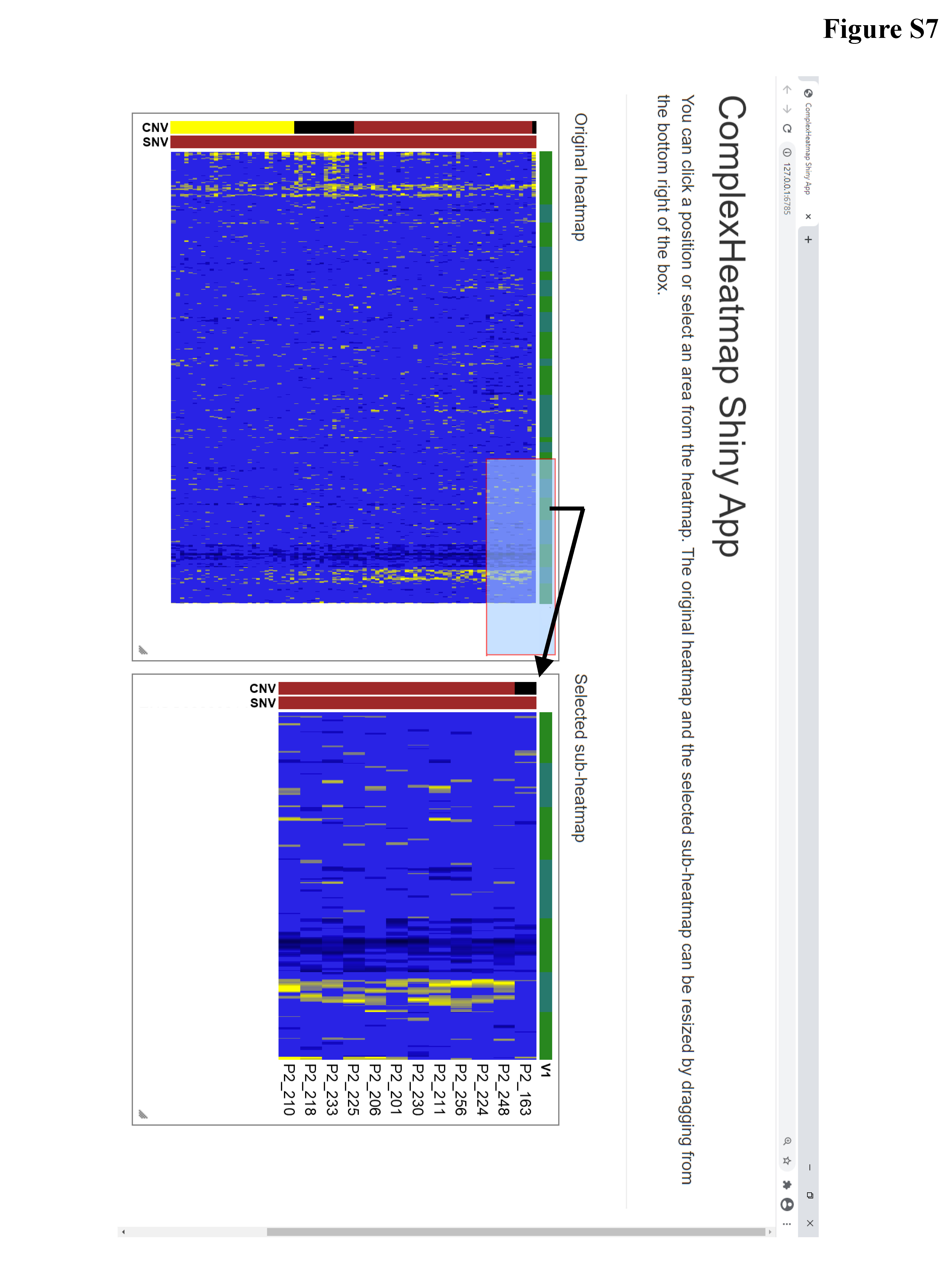

Supplement: Supplementary file 7 — Additional file 7: Figure S7. The screen shot of interactively examination of the identified clones with Shiny app. [file 12859_2022_4625_MOESM7_ESM.jpg]
